# Supplementary material for: MHC Class I-Restricted TCR-Transgenic CD4+ T Cells Against STEAP1 Mediate Local Tumor Control of Ewing Sarcoma In Vivo
Source: Cells. 2020 Jun 29;9(7):1581. doi: 10.3390/cells9071581 (PMC7408051; doi:10.3390/cells9071581)
Supplement: Supplementary file 1 [file cells-09-01581-s001.pptx]

## Slide 1
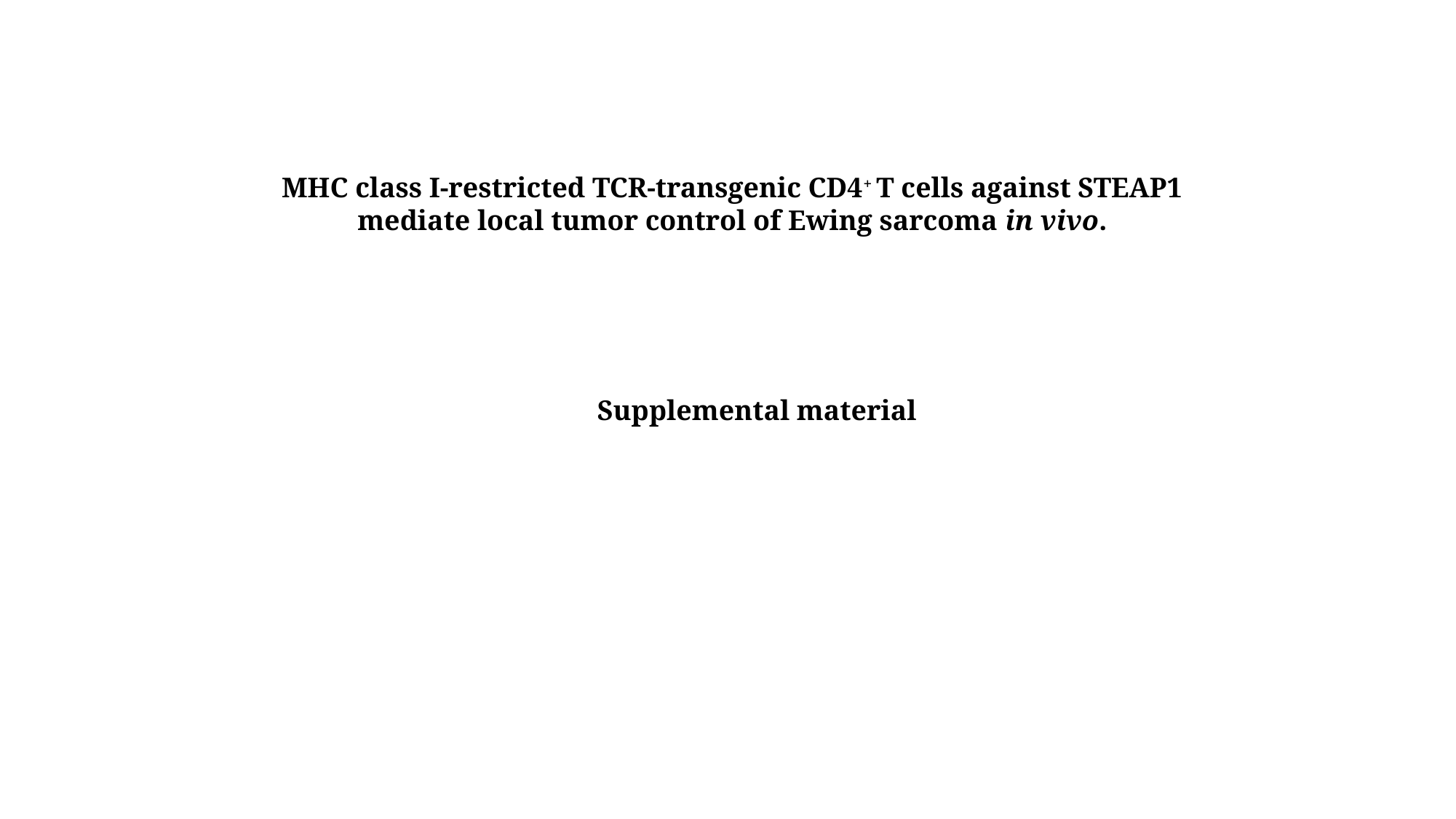

MHC class I-restricted TCR-transgenic CD4+ T cells against STEAP1
mediate local tumor control of Ewing sarcoma in vivo.
Supplemental material

## Slide 2
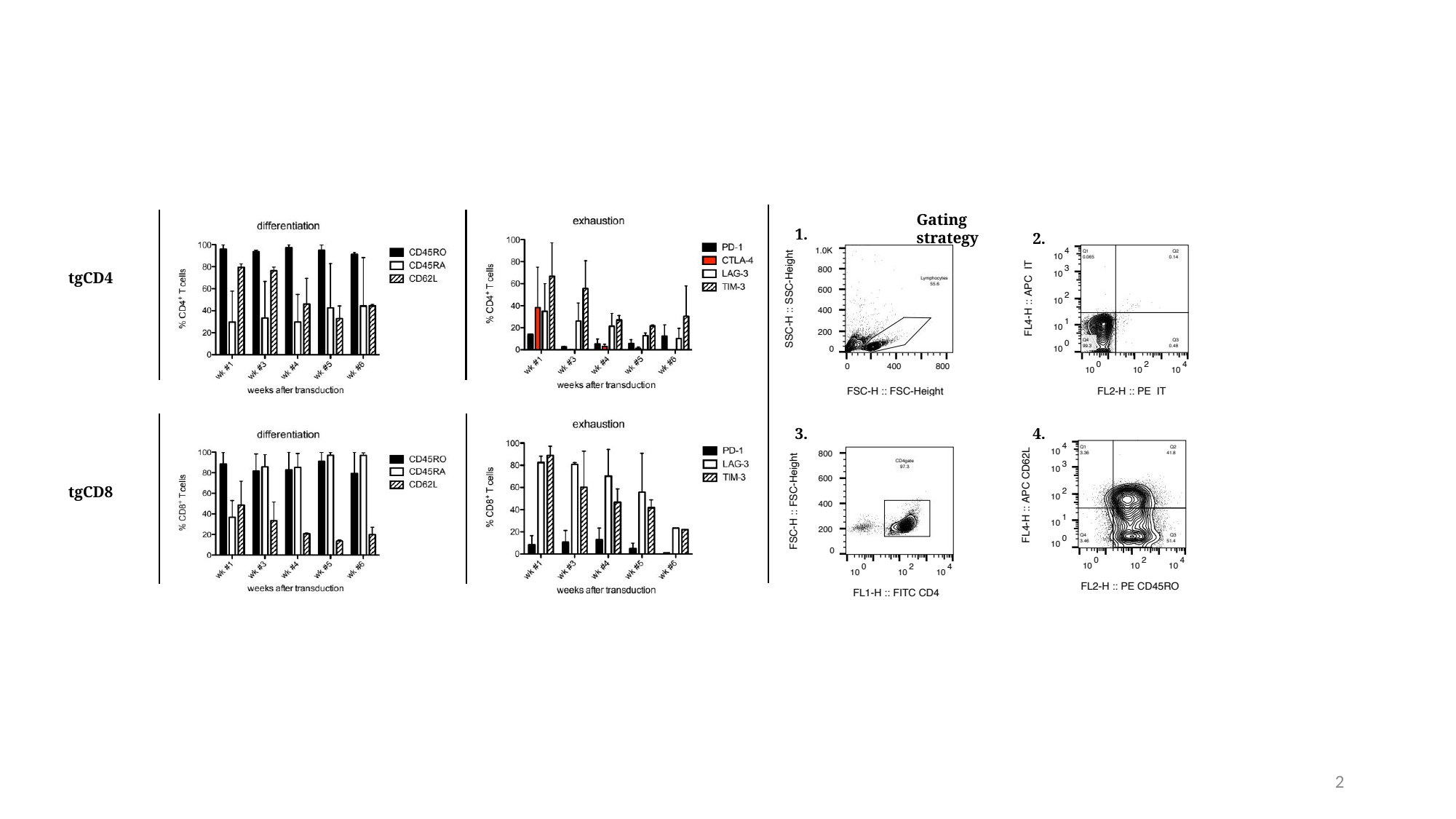

Gating strategy
1.
2.
tgCD4
3.
4.
tgCD8
2

## Slide 3
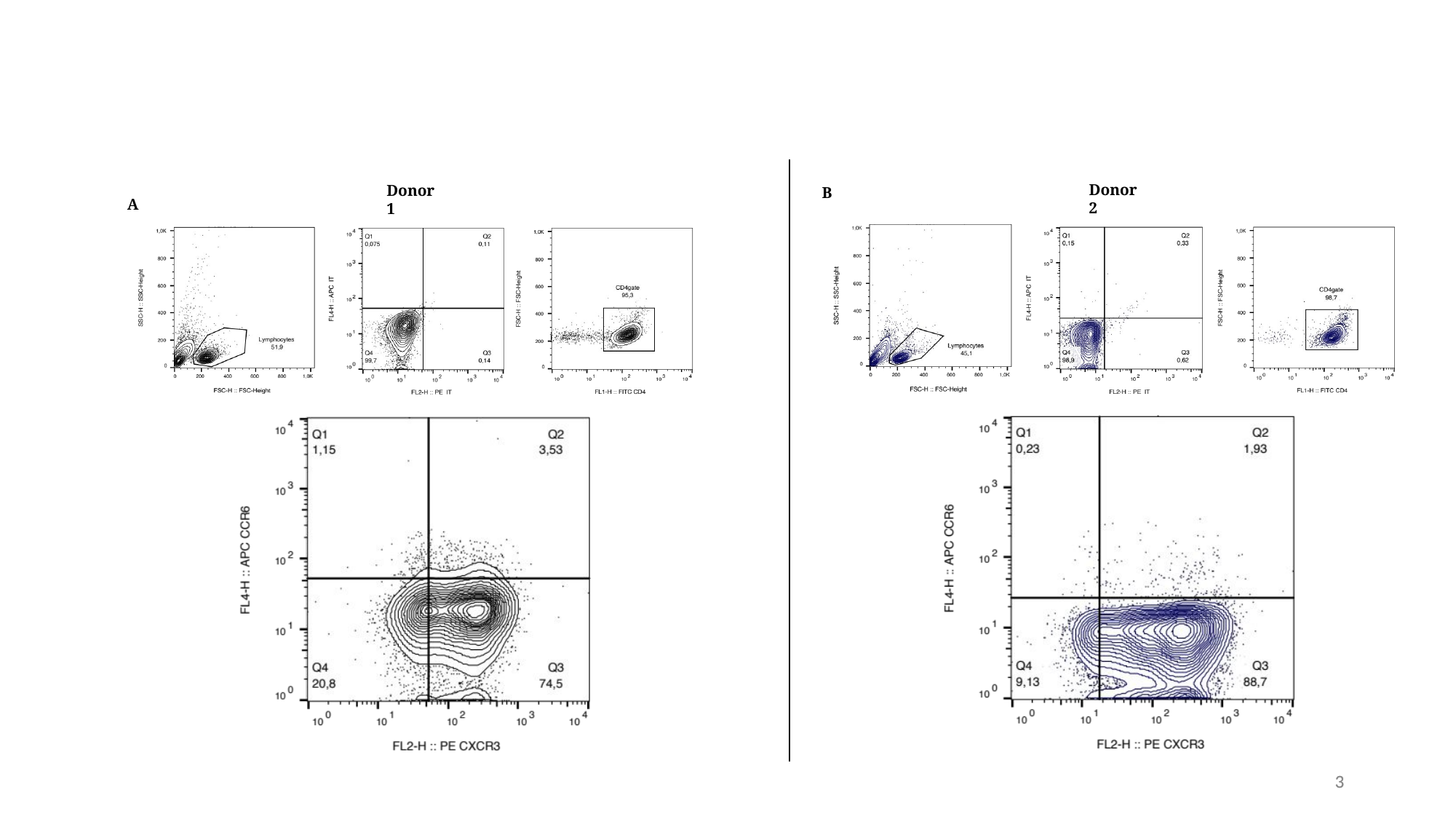

Donor 2
Donor 1
B
A
3
3

## Slide 4
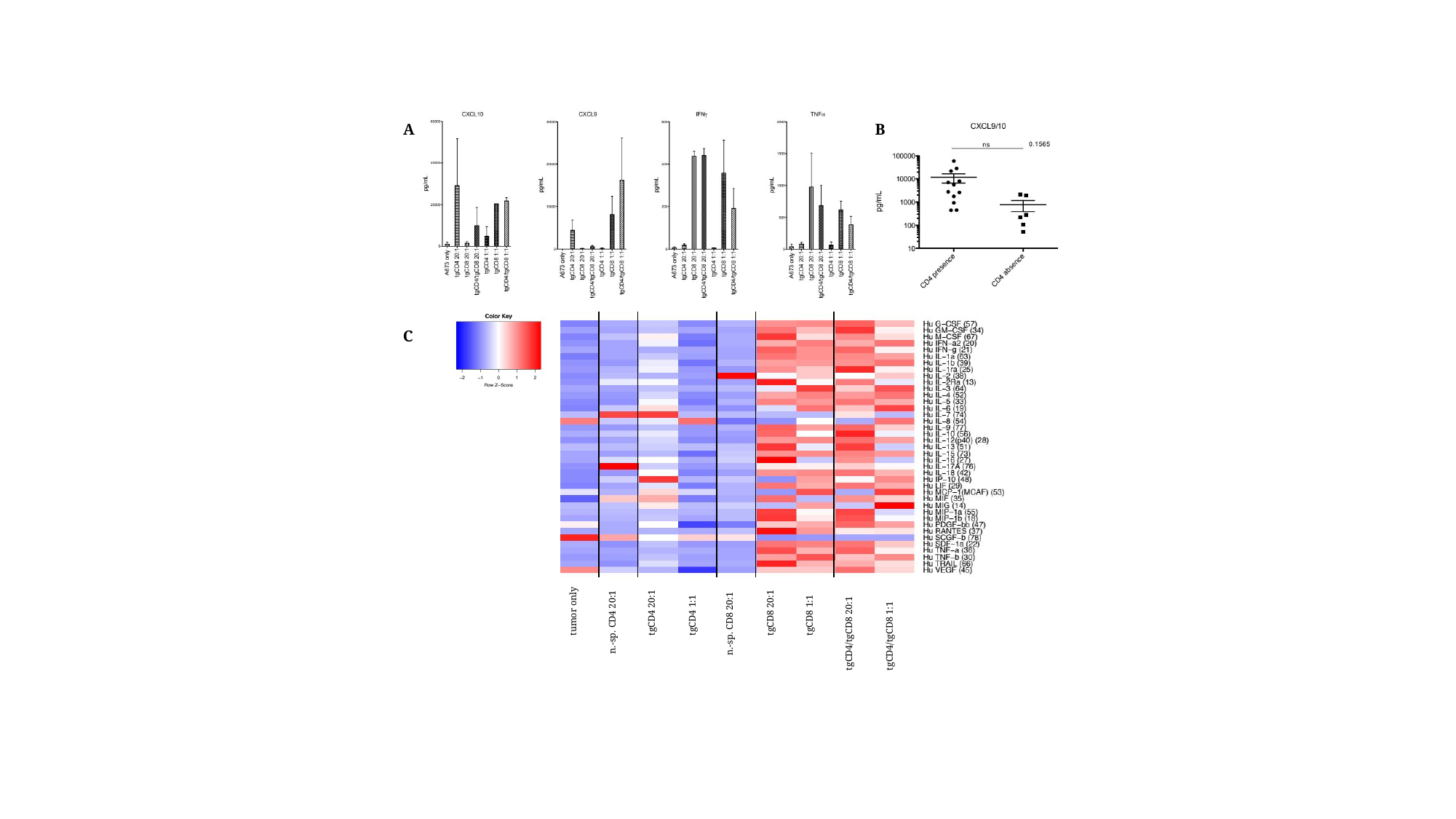

A
B
tumor only
tgCD4 1:1
tgCD4 20:1
tgCD8 20:1
tgCD8 1:1
n.-sp. CD4 20:1
n.-sp. CD8 20:1
tgCD4/tgCD8 1:1
tgCD4/tgCD8 20:1
C

## Slide 5
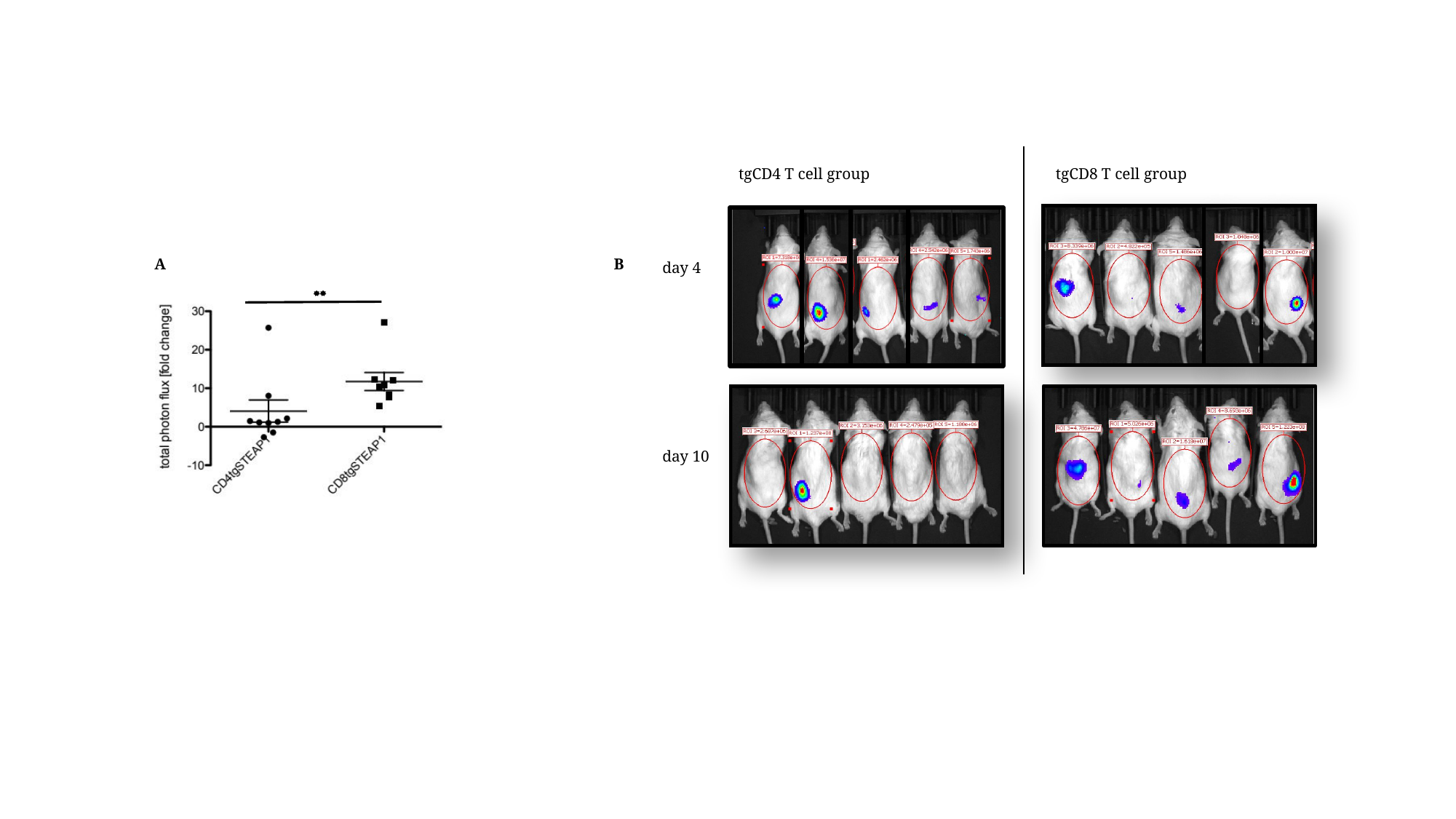

tgCD4 T cell group
tgCD8 T cell group
day 4
day 10
A
B

## Slide 6
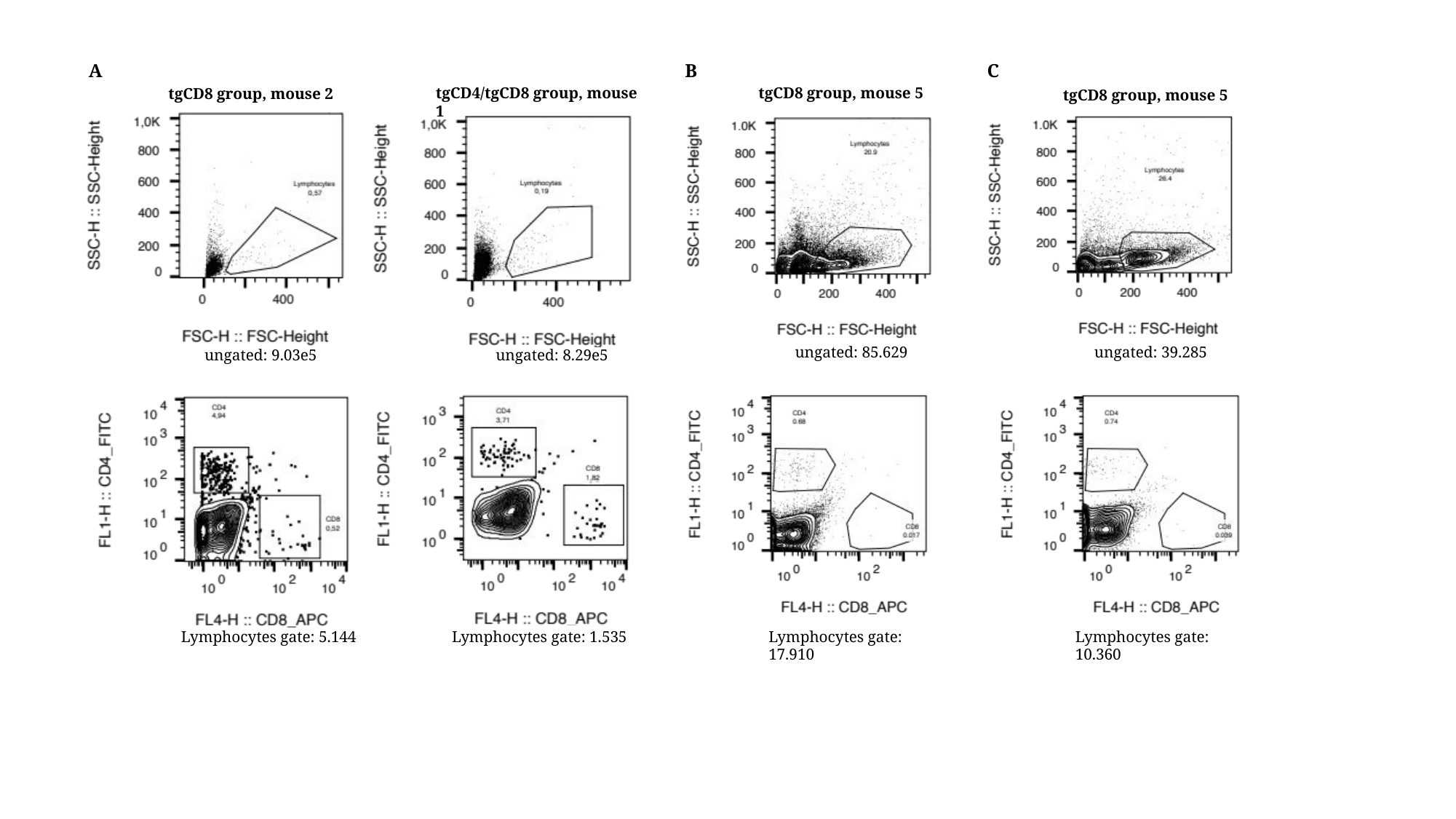

A
B
C
tgCD4/tgCD8 group, mouse 1
tgCD8 group, mouse 5
tgCD8 group, mouse 2
tgCD8 group, mouse 5
ungated: 85.629
ungated: 39.285
ungated: 9.03e5
ungated: 8.29e5
Lymphocytes gate: 10.360
Lymphocytes gate: 5.144
Lymphocytes gate: 1.535
Lymphocytes gate: 17.910

## Slide 7
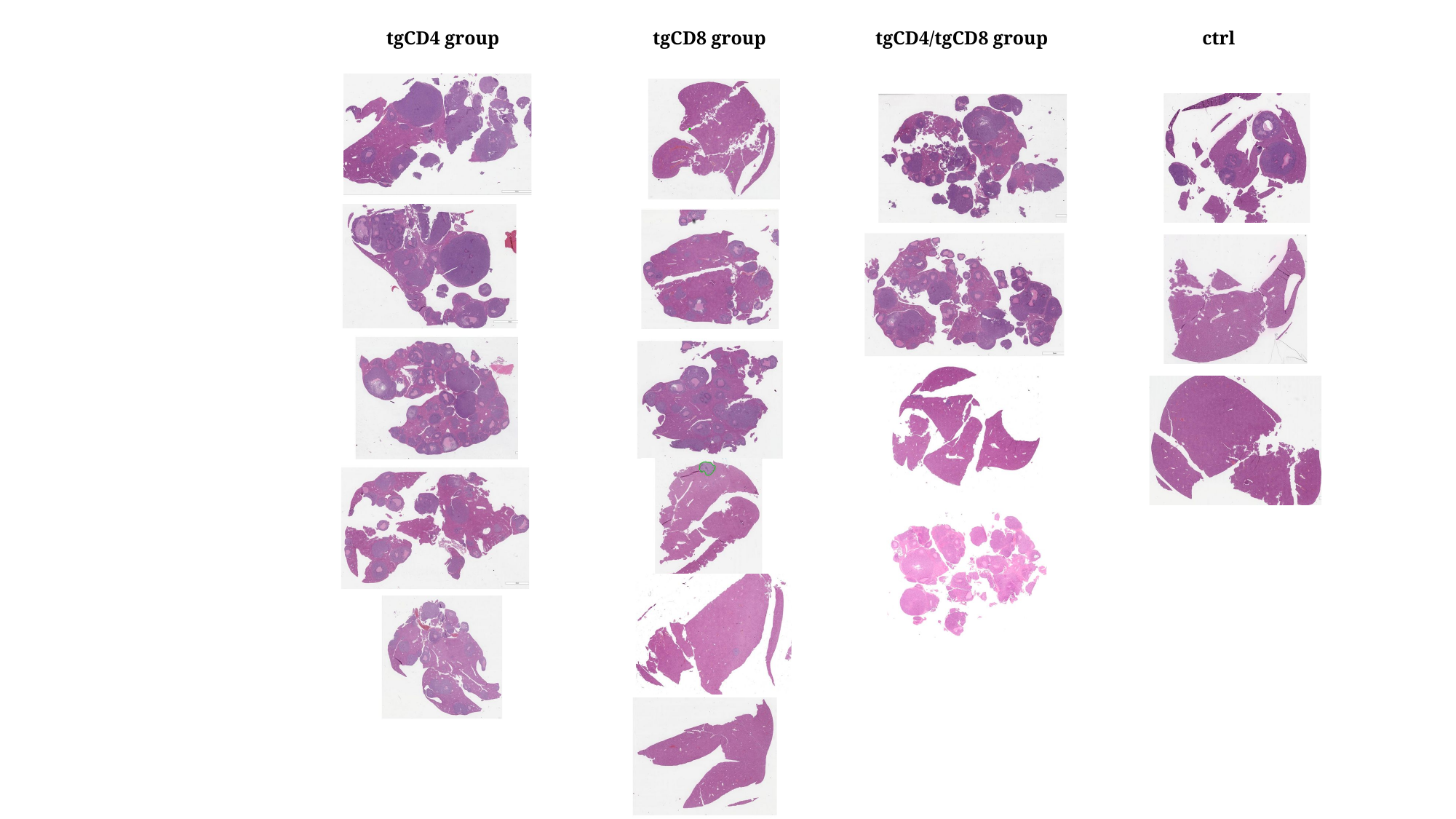

tgCD4/tgCD8 group
ctrl
tgCD4 group
tgCD8 group

## Slide 8
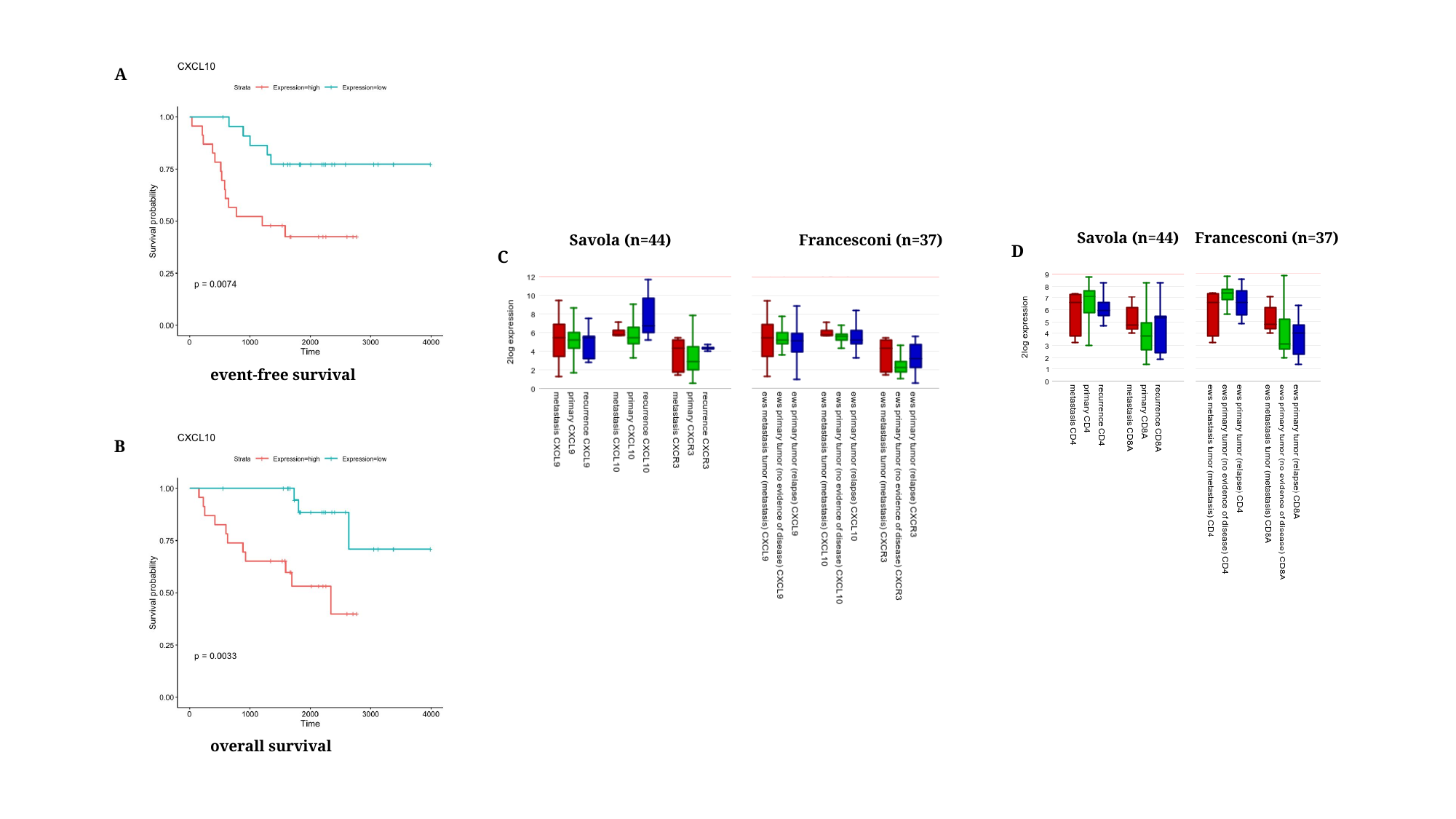

A
Francesconi (n=37)
Savola (n=44)
Savola (n=44)
Francesconi (n=37)
D
C
event-free survival
B
overall survival
